# The Phase Relations of the Co-Ni-In Ternary System at 673 K and 873 K and Magnetic Properties of Their Compounds

**DOI:** 10.3390/ma13183990

**Published:** 2020-09-09

**Authors:** Tonghan Yang, Wei He, Guojian Chen, Weijing Zeng, Jinzhi Wang, Lingmin Zeng, Jianlie Liang

**Affiliations:** 1 College of Chemistry and Chemical Engineering, Guangxi University, Nanning 530004, China; yangthan199@163.com; 2 School of Resources, Environment and Materials and Guangxi Key Laboratory of Processing for Non-ferrous Metallic and Featured Materials, Guangxi University, Nanning 530004, China; chgjxx@163.com (G.C.); zmszengweijing@163.com (W.Z.); lmzeng@gxu.edu.cn (L.Z.); 3 School of Materials and Chemical Engineering, Ningbo University of Technology, Ningbo 315211, China; wangjz@nbut.edu.cn; 4 School of Science, Guangxi University of Nationalities, Nanning 530006, China; liangjl1971@126.com

**Keywords:** Co-Ni-In, phase diagram, X-ray diffraction, magnetic properties

## Abstract

The phase relationships of the ternary Co-Ni-In system at 673 K and 873 K were investigated by means of powder X-ray diffraction, scanning electron microscopy equipped with energy dispersive spectroscopy, and optical microscopy. Though CoIn_2_ does not exist at 873 K, the ternary solid solution Co_1−x_Ni_x_In_2_ exists at both 673 K and 873 K with different composition ranges. The Rietveld refinements were carried out to investigate the crystal structure of Co_1−x_Ni_x_In_2_ (x = 0.540, and 0.580) and Ni_2−x_Co_x_In_3_ (x = 0.200). The magnetization dependence of temperature (MT) curves of Ni_2−x_Co_x_In_3_ (x = 0.200) and Co_1−x_Ni_x_In_2_ (x = 0.540) are similar to those of the ferromagnetic shape memory alloys Ni-Mn-A (A = Ga, Sn, and In), but do not undergo martensitic transformation. The maximum magnetic entropy changes in Ni_2−x_Co_x_In_3_ (x = 0.200) and Co_1−x_Ni_x_In_2_ (x = 0.540) under 3T are 1.25 and 1.475 J kg^−1^K^−1^, respectively.

## 1. Introduction

Recently, Ni-Mn-In has drawn increasing attention due to its fascinating multifunctional properties including its shape memory effect [1], magnetocaloric effect [2], elastocaloric effect [3], magnetothermal conductivity [4], magnetic superelasticity [5], barocaloric effect [6], and large exchange bias effect [7] associated with the martensitic-type phase transformation. A large number of researches have shown that the properties of the Ni-Mn-In alloys have been highly improved when accompanied with a small amount of Co doping into Ni-Mn-In ternary compounds [8,9,10]. This brings more attention to the Ni-Co-Mn-In quaternary compounds and another upsurge of research of the alloys [11,12,13,14]. The martensitic transformation, which belongs to first-order magnetostructural transformation, led to a large magnetic entropy change (ΔS) which makes Ni-Co-Mn-In alloys promising candidates for magnetic refrigeration materials [15,16,17]. A giant magnetocaloric effect driven by structural transitions was found in Ni_45_._7_Mn_36_._3_In_13_Co_5_, which resulted in a high adiabatic temperature change (ΔT_ad_) of −6.2 K under a low field of 2T [2]. In Cheng’s work, the temperature-induced martensitic transformation of Ni_42_Co_8_Mn_37_._7_In_12_._3_ alloy achieved a giant (ΔS) of 14.30 J·K^−1^·kg ^−1^ and refrigeration capacity (RC) up to 549 J·K^−1^. Additionally, the near-room-temperature working temperature range of 248–295 K makes it superior among a number of reported magnetocaloric materials [18]. Large magnetic field-induced martensitic transformation led to enormous recoverable deformation, which can be easily observed in Ni-Co-Mn-In alloys, making it an attractive shape memory functional material [19,20,21]. Stresses of over 100 MPa were generated in Ni_45_Co_5_Mn_36_._7_In_13_._3_ on the field of 7 T. 3 % deformation, and full recovery of the original shape was discovered as a result of magnetic field-induced strains [1]. After sintering at 873 K, Ni_45_Mn_36_._6_In_13_._4_Co_5_ showed an almost perfect shape memory effect in which martensitic transformation played the dominant role, since the shape recovery was as high as 11.4% [22]. With such excellent magnetocaloric and shape memory properties, they can extensively serve society. However, large thermal transformation hysteresis, needing a high-driving magnetic field and poor mechanical properties, severely hinders their commercialization [10,23,24]. Phase diagrams are important for designing and preparing of Ni-Co-Mn-In alloys with potentially excellent properties. In addition, they enable the exploration of new functional materials. Only the phase diagrams of the Ni-Mn-In and Ni-Co-Mn ternary systems among the four sub-ternary systems of the Ni-Co-Mn-In quaternary system have been studied [25,26,27,28], while the phase diagrams of the other systems are not available in literature. At the same time, investigation on the magnetic properties of the compounds in these three ternary systems also plays a significant role in exploring new potential magnetic materials, such as refrigeration materials. This paper will focus on one of the four sub-ternary systems of the Ni-Co-Mn-In quaternary system, the Co-Ni-In system, and the magnetic properties of its compounds.

The phase diagrams of the binary systems Co-Ni, Co-In, and Ni-In related to the Ni-Co-In ternary system have been studied [29,30,31]. According to the Co-Ni binary phase diagram, there is no intermediate phase in this system. Co and Ni form an infinite solid solution that exists in the form of α-(CoNi) (space group: Fm$\bar{3}$m) above 695 K. Below 695 K, the alloys are in a ε-(CoNi) hexagonal phase (Space group P6_3_/mm) at a low nickel content range. With the increase in Ni content, the ε-Co→α-Co transformation occurs. Two binary compounds, CoIn_2_ and CoIn_3_, exist in the Co-In binary system, and the compounds are formed by peritectic reactions of α-Co + L → CoIn2 (823 K) and CoIn2 + L→ CoIn3 (763 K), respectively. The binary system undergoes a monotectic reaction at 1559 K. The composition of the monotectic is about 77 at.% In. 

There are seven binary compounds, i.e., Ni_3_In, Ni_2_In, ζ, Ni_13_In_9_, NiIn, δ, Ni_2_In_3_, and Ni_3_In_7_ in the Ni-In binary system according to Ref. [31]. Intermetallic compounds of Ni_3_In, Ni_2_In, NiIn, Ni_2_In_3_, and Ni_3_In_7_ are stoichiometrically determined. The ζ phase is stable from 755 to 1223 K with a maximum range from 31.2 to 40.5 at.% In, and the compound Ni_13_In_9_ has a composition range of 38.0–41.0 at.% In, and the compound Ni_13_In_9_ have a composition range of 38.0–41.0 at.% In. Additionally, the δ phase forming at higher temperature is stable in the temperature range of 1043–1203 K and has a composition range of 52–58.2 at.% In. The solid solubility of In in Ni increases with temperature, which increases from 3.3 to 13.4 at.% In when temperature increases from 693 to 1181 K. No solubility of Ni in In has been reported. A ternary compound, Ni_2_CoIn, was investigated theoretically by first principles in [32], showing that the compound crystallized in a cubic structure with a space group of Fm$\bar{3}$m and lattice parameter a = 0. 5944 nm. The Ni atom occupies the 8c (0.25, 0.25, 0.25) site, Co occupies the 4b (0.5, 0.5, 0.5) site, and the In atom occupies the 4a (0, 0, 0) site. Calculation of formation energy indicates the instability of the austenitic structure in Ni_2_CoIn and a strong tendency of martensitic transformation. Table 1 shows the crystallographic data of the compounds in the Co-Ni-In system.

## 2. Experimental

All samples with a mass of 2 g were prepared in an electric arc furnace under argon atmosphere. The purities of initial metals Co, Ni, and In were 99.9 wt. %, 99.99 wt. % and 99.9 wt. %, respectively. The material source is General Research Institute for Nonferrous Metals (Beijing, China). A proper amount of In was added to each samples due to the loss of In. Titanium was used as the oxygen scavenger during the melting process. The samples were melted three times for the purpose of homogeneity of the alloy samples. All the as-cast samples were sealed in evacuated quartz tubes to anneal at higher temperature for further homogenization. The In-rich (> 50 at.% In) alloys were kept at 923 K/673 K for 30 days, while the other alloys homogenized at 1073 K for 20 days. After homogenization annealing, the samples used for the 873 K section were cooled down to 873 K with a cooling rate of 50 K/day and maintained at 873 K for 20 days to reach equilibrium, while those used for the 673 K section were cooled down to 673 K and maintained at 673 K for 20 days. Finally, all the samples were quenched into ice-water mixture.

The X-ray powder diffraction (XRD) data of the Co-Ni-In alloy samples were obtained by using a Rigaku D/max 2500 V powder diffractometer (Cu Kα1 radiation, λ = 1.54060 Å; Tokyo, Japan). The powder XRD data for phase analysis were collected in a continuous scanning mode, and those for Rietveld refinement were collected in a step scanned mode with a step size of 0.02°. The high temperature XRD data were collected by using a Bruker D8 advance diffractometer. The Rietveld refinements for some selected samples were carried out by using FullProf programs [34,35]. Scanning electron microscopy (SEM, Hitachi S-3400N or SU8000; Tokyo, Japan) and optical microscopy (Axio Imager A2m, Zeiss, Jena, Germany) were used for microstructure analysis, and energy dispersive analysis (EDS) was applied for the measurements of sample chemical and phase compositions. The samples used for SEM/EDS measurements were corroded with clear water. Magnetic measurements were carried out in the Physical Property Measurement System (PPMS, Quantum Design; San Diego, CA, USA).

## 3. Results and Discussion

The phase analysis was performed on all the XRD data of the equilibrated Co-Ni-In samples with the aid of the Powder Diffraction File 2 (PDF2) database released by International Center for Diffraction Data (ICDD). The diffraction patterns of those compounds absent in the PDF2 database were calculated from the crystallographic data available in references. By carrying out the phase identification on the XRD patterns of each Co-Ni-In sample, the phase components of each sample were obtained. The selected samples were further observed by SEM/EDS and optical microscopy for phase identification and composition measurements.

### 3.1. Phase Analysis

#### 3.1.1. Phase Analysis at 673 K

**Figure 1:**
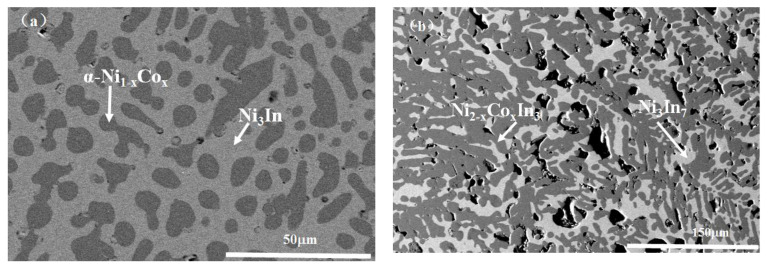
BSE micrograph of (**a**) No. 1 (Co10Ni75In15) and (**b**) No. 2 (Co4Ni34In62).

Table S1 shows the XRD and SEM/EDS analysis results of the selected Co-Ni-In samples at 673 K. The analysis on the XRD patterns of all the binary and ternary Co-Ni-In samples shows eight binary compounds, i.e., Ni_3_In, Ni_2_In, Ni_13_In_9_, NiIn, Ni_2_In_3_, Ni_3_In_7_, CoIn_3_, and CoIn_2_, existing at 673 K. The binary compounds Ni_4_In and Ni_13_In_7_ were not observed in our experimental conditions. This is in good agreement with the Co-Ni, Co-In, and In-Ni binary phase diagrams. The Backscattered electrons (BSE) micrographs of the alloys No. 1 (Co10Ni75In15) and No. 2 (Co4Ni34In62) are shown in Figure 1a,b. Both alloys contain two phases. EDS analysis on alloy No. 24 (Co10Ni75In15) in Figure 1a revealed that the dark gray phase with composition of Co35.53(5)Ni62.15(4)In2.31(4) was identified as α-Ni Co_x_ (x = 0.355), and the gray phase with composition of Co1.02(4)Ni72.26(5)In26.72(5) was Ni_3_In. In Figure 1b, for the sample No. 25 (Co4Ni34In62), the dark gray phase with composition of Co 2.36(4)Ni59.12(3)In38.52(5) was verified to be Ni_2 – x_Co_x_In_3_ (x = 0.118), and the gray phase with composition of Co0.86(6)Ni32.43(5)In66.72(6) was Ni_3_In_7_. These results proved the existence of Ni_3_In, Ni_2_In_3_, and Ni_3_In_7_ at 673 K, which is in agreement with the literature [26,31].

**Figure 2:**
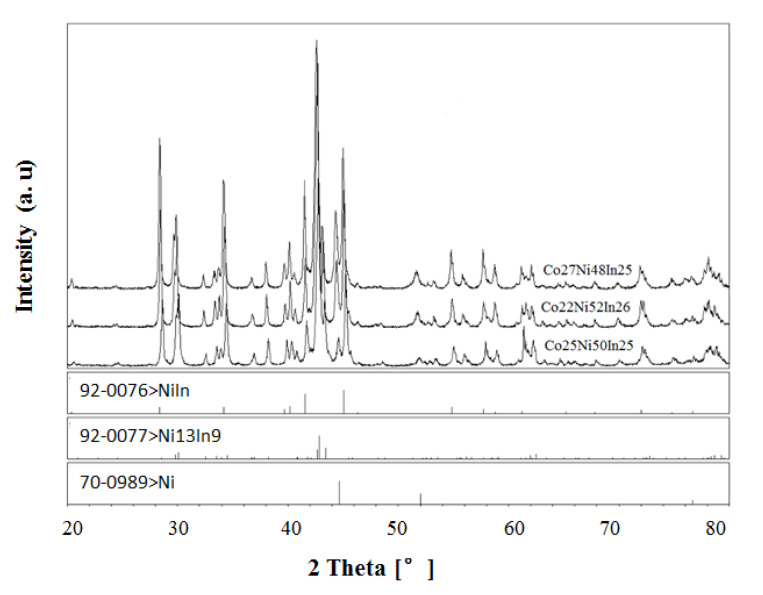
XRD patterns of samples No. 3 (Co25Ni50In25), No. 4 (Co22Ni52In26), and No. 5 (Co27Ni48In25).

**Figure 3:**
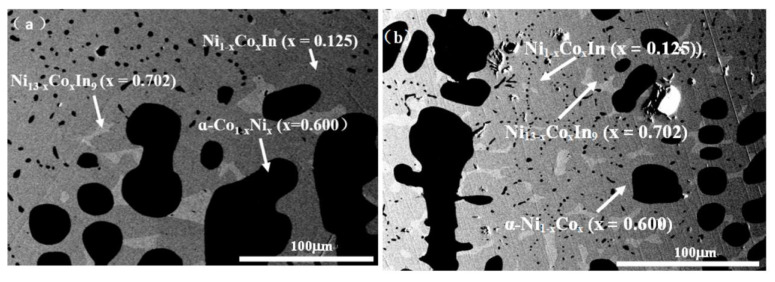
BSE micrograph of samples (**a**) No. 3(Co25Ni50In25) and (**b**) No. 4 (Co22Ni52In26).

In order to verify the existence of the ternary compound Ni_2_CoIn, which was investigated by using the first principle calculation as described by Bai et al. [35], a series of samples with compositions near Ni_2_CoIn was prepared. Figure 2 presents the XRD patterns of sample No. 3 (Co25Ni50In25), No. 4 (Co22Ni52In26), and No. 5 (Co27Ni48In25), and none of the XRD data of these samples correspond to the diffraction pattern calculated from the crystallographic data of Ni_2_CoIn as shown in the literature [35]. The XRD analysis on all of these alloys showed that these alloys contained the three phases of Ni_13 − x_Co_x_In_9_ (x = 0.702), Ni_1−x_Co_x_In (x = 0.125), and α-Ni_1−x_Co_x_ (x = 0.400) pointing to the absence of Ni_2_CoIn at 673 K. Figure 3a,b show the SEM micrographs of samples No. 5 and No. 6, respectively, as well as the composition of each phase obtained by EDS. The results also indicated that these two alloys contained the three phases of Ni_13 − x_Co_x_In_9_ (x = 0.702), Ni_1−x_Co_x_In (x = 0.125) and α-Ni_1−x_Co_x_ (x = 0.4), which proved the non-existence of Ni_2_CoIn at 673 K.

#### 3.1.2. Phase Analysis at 873 K

Table S2 gives the XRD and SEM/EDS analysis results of the selected Co-Ni-In samples at 873 K. The analysis on the XRD patterns of all the binary and ternary Co-Ni-In samples shows that six binary compounds, i.e., Ni_3_In, Ni_2_In, Ni_13_In_9_, ξ, NiIn, and Co_1−x_Ni_x_In_2_, exist at 873 K.

**Figure 4:**
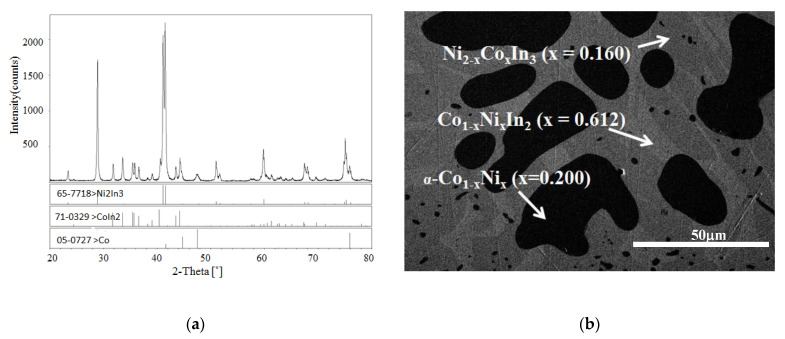
(**a**) XRD pattern and (**b**) BSE micrograph of No. 26 (Co28Ni24In48).

**Figure 5:**
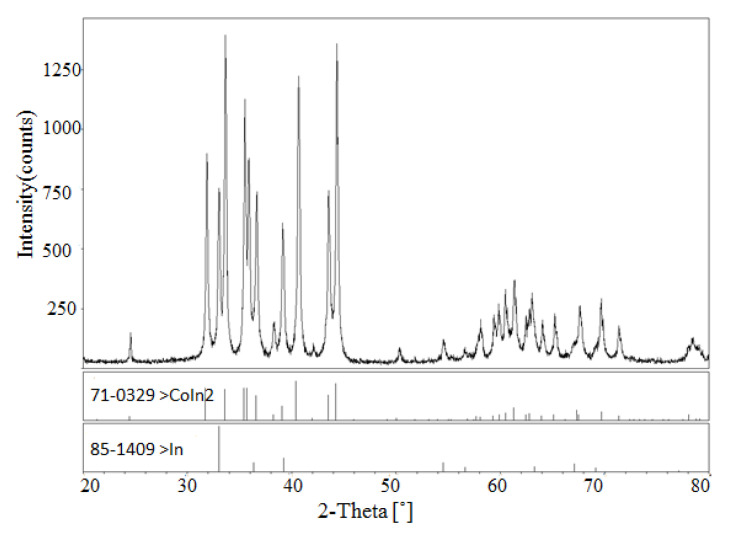
XRD pattern of No.33 (Co26Ni6In68).

According to [12], a peritectic reaction L + (α) Co → CoIn_2_ occurs at 823 K, and the compound CoIn_2_ does not exist at 873 K. However, in the present work, a solid solution Co_1−x_Ni_x_In_2_ was found at 873 K. The solid solution of Co_1−x_Ni_x_In_2_ crystallized in the same crystal structure as that of CoIn_2_. Figure 4a,b show the XRD pattern and SEM micrograph of alloy No. 26 (Co28Ni24In48). The XRD analysis result revealed that the alloy contained the three phases of Co_1−x_Ni_x_In_2_ (x = 0.612), Ni_2−x_Co_x_In_3_ (x = 0.450), and α-Co_1−x_Ni_x_ (x = 0.200), as seen in Figure 4a. The composition measurement showed that the grey phase with composition of Co8.69(5)Ni32.99(4)In58.32(4), the light gray phase with composition of Co20.10(5)Ni16.82(6)In63.08(5), and the dark phase with composition of Co82.21(5)Ni17.16(6)In0.63(5) were identified to be the three phases of Ni_2−x_Co_x_In_3_ (x = 0.160), Co_1−x_Ni_x_In_2_ (x = 0.612), and α-Co_1−x_Ni_x_ (x = 0.200), respectively, as seen in Figure 4b. Figure 5 shows the XRD pattern of alloy No. 27 (Co26Ni6In68). The XRD pattern in Figure 5 clearly indicates that the alloy contained the two phases of Co_1−x_Ni_x_In_2_ and In, which confirmed Co_1−x_Ni_x_In_2_ existed at 873 K once again. This suggests that the addition of Ni into CoIn_2_ stabilized the compound and raised the temperature of the peritectic reaction L + (α) Co → Co_1−x_Ni_x_In_2_.

**Figure 6:**
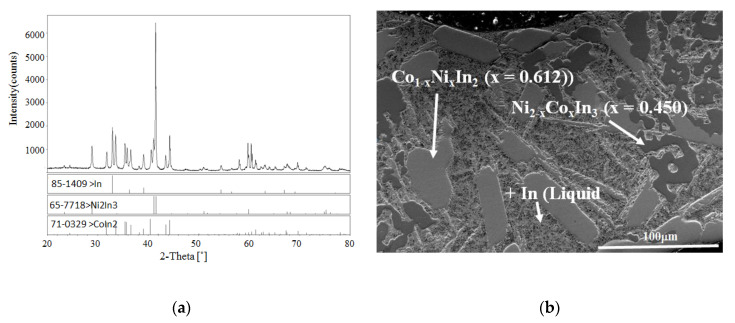
(**a**) XRD pattern and (**b**) BSE micrograph of No. 32 (Co8Ni22In70).

**Figure 7:**
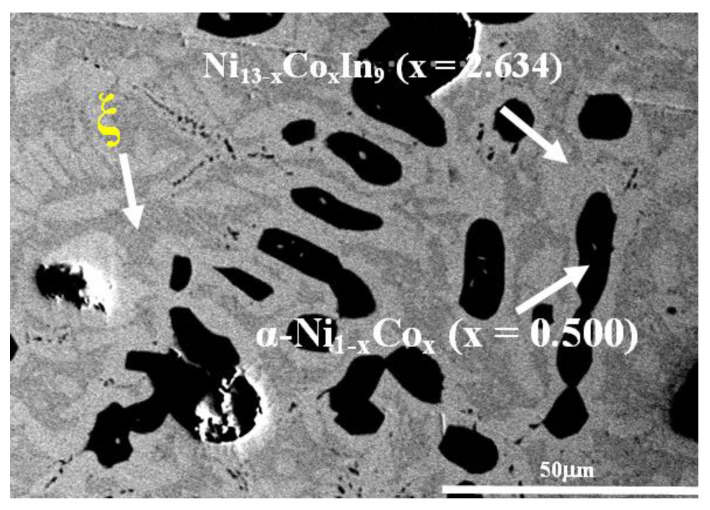
BSE micrograph of No. 28 (Co18Ni52In30).

The XRD pattern and SEM micrograph of alloy No. 28 (Co8Ni22In70) are shown in Figure 6a,b, respectively. The XRD analysis of the alloy indicated that the three phases of Co_1 – x_Ni_x_In_2_ (x = 0.612), Ni_2 – x_Co_x_In_3_ (x = 0.450) and In (Liquid) coexisted in the alloy, and no diffraction patterns of Ni_3_In_7_ and/or CoIn3 were observed. The SEM/EDS analysis on alloy No.28 also gave the same results. This indicates that Ni_3_In_7_ and CoIn_3_ do not exist at 873 K. Figure 7 shows the SEM micrograph of No. 29 (Co18Ni52In30) alloy. It is clearly seen that the alloy is composed of three phases. EDS measurements showed that the gray phase with composition of Co10.91(5)Ni46.53(4)In42.56(4) was identified as Ni_13 − x_Co_x_In_9_ (x = 2.634), the dark gray phase with composition of Co5.94(6)Ni55.83(5)In38.28(5) was verified to be ξ, and the dark phase with composition of Co48.69(4)Ni49.12(4)In2.19(3) was α-Ni_1−x_Co_x_ (x = 0.500). This result suggests that the ξ phase existed at 873 K, which is similar to Schmetterer’s investigation [36].

Compared to the phases at 673 K, the binary compounds, i.e., Ni_3_In, Ni_2_In, Ni_13_In_9_, NiIn, Ni_2_In_3_, and ξ, exist at 873 K, while the binary compounds Ni_3_In_7_, CoIn_3_, and CoIn_2_ disappeared at 873 K. Although CoIn_2_ does not exist at 873 K, the ternary solid solution Co_1−x_Ni_x_In_2_ exists at both of 673 K and 873 K with different composition ranges. No new binary and ternary compounds were found at 673 K and 873 K.

### 3.2. Solid Solubility

The solid solubilities of Co In Ni_2_In, NiIn, N_13_In_9_, Ni_2_In_3_, and Ni, as well as Ni in CoIn_2_ and Co at 673 K and 873 K were determined by XRD using the phase-disappearing and lattice parameter method combined with the SEM (EDS). The rough maximum solid solubility of above compounds was estimated by comparing the movement of the diffraction patterns of the single phases to the disappearance of the phases. A few series samples such as Ni_1−x_Co_x_In and Co_1−x_Ni_x_In_2_ were prepared for the purpose of the solid solubility determination in the Co-Ni-In ternary system. The computer software Jade 5.0 was used to calculate and refine the lattice parameters of the samples Ni_1−x_Co_x_In and Co_1−x_Ni_x_In_2_ from the XRD patterns.

#### 3.2.1. Solid Solubility at 673 K

**Figure 8:**
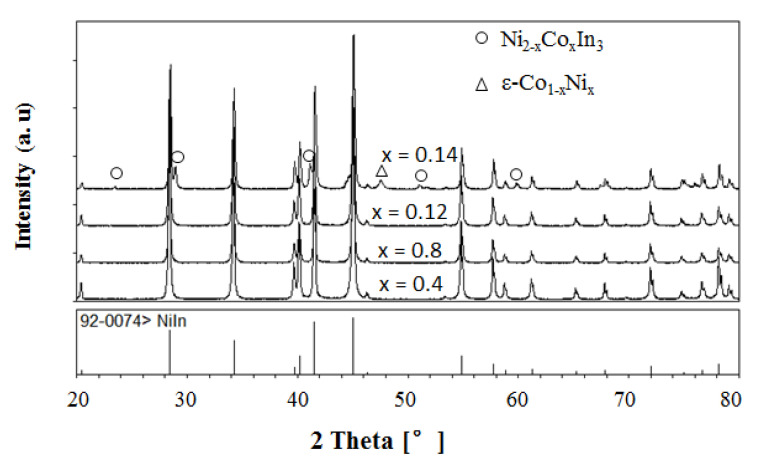
XRD patterns of the sample Ni_1−x_Co_x_In (x = 0.04, 0.08, 0.12, 0.14).

**Figure 9:**
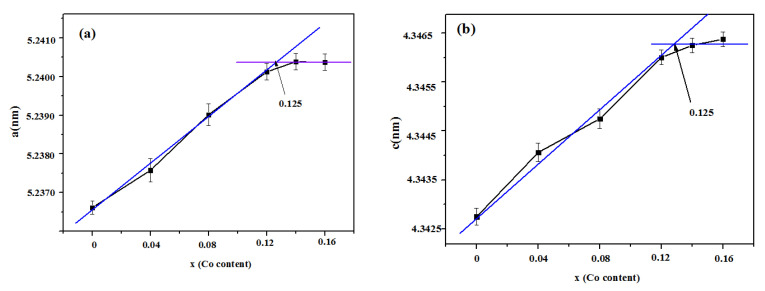
Variation of (**a**) the lattice parameter a and (**b**) lattice parameter c of Ni_1−x_Co_x_In on Co content.

**Figure 10:**
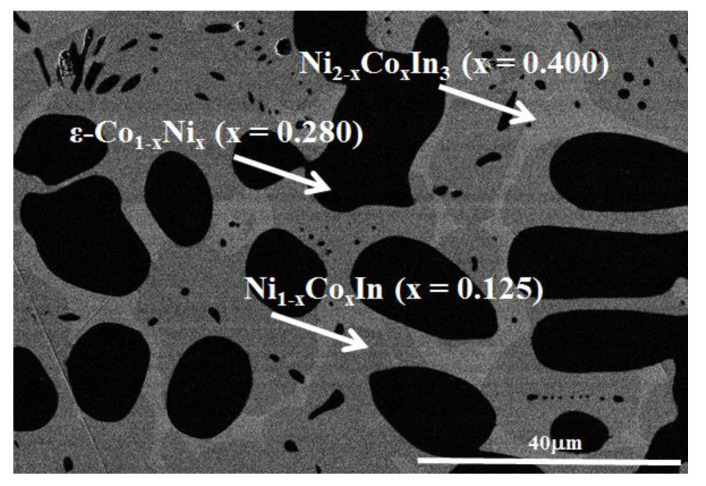
BSE micrograph of the sample Ni_1−x_Co_x_In (x = 0.14).

Figure 8 presents the XRD patterns of the samples Ni_1−x_Co_x_In (x = 0.04, 0.08, 0.12, 0.14) at 673 K. It can be clearly seen that these samples (except that of x = 0.14) contained the single phase of Ni_1−x_Co_x_In, pointing to the maximum solid solubility of Co in NiIn being between x = 0.12 and x = 0.14. Figure 9a,b show the variation in the lattice parameter a and the lattice parameter c of Ni_1−x_Co_x_In with the content of Co, which were calculated from the XRD patterns by Jade 5.0. It can be seen from Figure 9a,b that the maximum solid solubility of Co in Ni_1−x_Co_x_In is x = 0.125 (6.25 at.% Co). Further analysis on the sample Ni_1−x_Co_x_In (x = 0.14) by the SEM (EDS) also showed that the alloy contains the three phases of Ni_1−x_Co_x_In (x = 0.125), Ni_2−x_Co_x_In_3_ (x = 0.400), and ε-Co_1−x_Ni_x_ (x = 0.280), as seen in Figure 10, which is felt in a three-phase region. The composition measurement shows that the dark grey phase with composition of Co5.75(5)Ni45.02(6)In49.23(6) is Ni_1−x_Co_x_In (x = 0.125), the grey phase with composition of Co7.82(4)Ni33.11(5)In58.07(5) is Ni_2−x_Co_x_In_3_ (x = 0.400) and the dark phase with composition of Co72.81(7)Ni26.18(6)In1.01(5) is ε-Co_1−x_Ni_x_ (x = 0.280). This caused the maximum solid solubility of Co in Ni_1−x_Co_x_In to be 5.82 at.% Co at 673 K, and this value is similar to that obtained by the lattice parameter method, i.e., x = 0.125 (6.25 at.% Co). This further supports that the maximum solid solubility of Co in Ni_1−x_Co_x_In is 6.25 at.% Co at 673 K.

Similarly, the maximum solid solubilities of Ni in Co_1−x_Ni_x_In_2_ and ε-Co_1−x_Ni_x_ were determined to be 18.64 and 28 at.% Ni at 673 K, respectively. The maximum solid solubilities of Co in Ni_2−x_Co_x_In, Ni_13 − x_Co_x_In_9_, Ni_2−x_Co_x_In_3_, and α-Ni_1−x_Co_x_ were about 3, 3.2, 8, and 60 at.% Co at 673 K.

#### 3.2.2. Solid Solubility at 873 K

**Figure 11:**
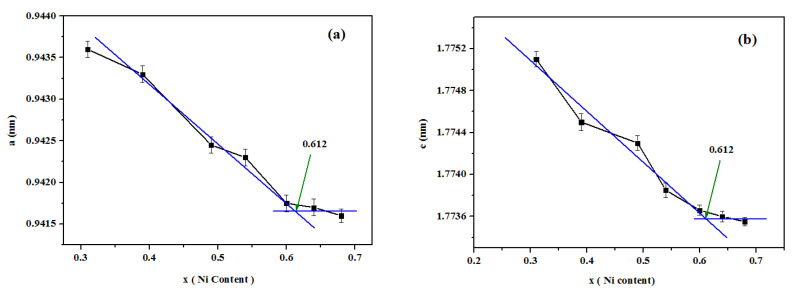
Variation of (**a**) the lattice parameter a and (**b**) lattice parameter c of Co_1−x_Ni_x_In_2_ on Ni content.

Figure 11a,b show the variation of the lattice parameter a and the lattice parameter c of Co_1−x_Ni_x_In_2_ with the content of Ni, which indicated that the maximum solid solubility of Ni in Co_1−x_Ni_x_In_2_ is x = 0.612 (about 20.19 at.% Co). The SEM micrograph of No. 26 is given in Figure 4b. The composition measurement of the light grey phase with composition of Co20.10(5)Ni16.82(5)In63.08(5), which was Co_1−x_Ni_x_In_2_ (x = 0.612), suggested that the maximum solid solubility of Co in Co_1−x_Ni_x_In_2_ was 20.1 at.% Co at 873 K. These two values are close. Although the binary CoIn_2_ is absent at 873 K, the addition of Ni in CoIn_2_ stabilized the compounds and kept the solid solution Co_1−x_Ni_x_In_2_ with a wide range appeared at 873 K. The solid solubility range of Co_1−x_Ni_x_In_2_ is 3-20.1 at.% Co at 873 K.

**Figure 12:**
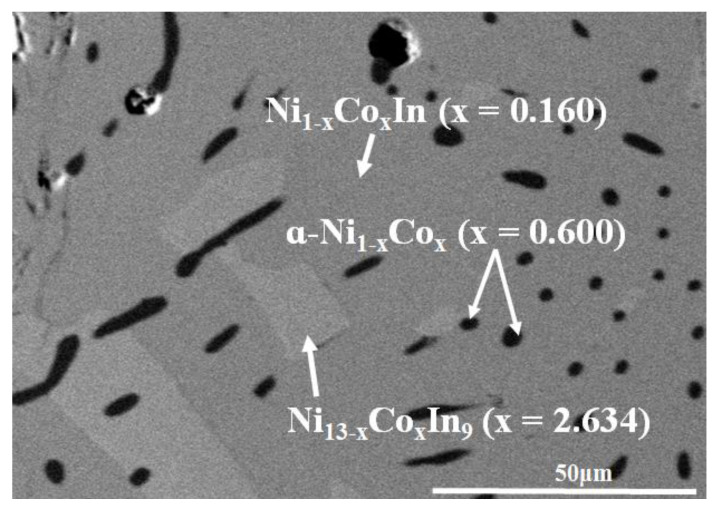
The BSE micrograph of No. 30 (Co25Ni45In35).

The SEM micrograph of alloy No. 30 (Co25Ni45In35) in Figure 12 clearly shows that the alloy contains three phases. Further composition measurements indicated that the grey phase with composition of Co7.43(5)Ni43.56(6)In49.01(6) was confirmed as Ni_1−x_Co_x_In (x = 0.160), the light grey phase with composition of Co12.03(6)Ni47.28(7)In40.69(6) was verified to be Ni_13 − x_Co_x_In_9_ (x = 2.634) and the dark phase with composition of Co61.21(5)Ni37.51(5)In1.28(5) was α-Ni_1−x_Co_x_ (x = 0.600). This further suggests that the maximum solid solubility of Co in Ni_13 − x_Co_x_In_9_ is about 12.03 at. % Co at 873 K.

Similarly, the maximum solid solubilities of Co In Ni_2−x_Co_x_In, Ni_13 − x_Co_x_In_9_, Ni_1−x_Co_x_In, Ni_2−x_Co_x_In_3_, and α-Ni_1−x_Co_x_ were found to be about 6, 12.03, 8, 9, and 60 at.% Co at 873 K, respectively. Both of the maximum solid solubilities of In in ε-Co_1−x_Ni_x_ and α-Ni_1−x_Co_x_ were observed to be less than 3 at.% In.

Clearly, temperature has a great effect on the solid solubility of the third element in the binary compounds of the Co-Ni-In ternary system. Normally, the maximum solid solubilities of the third element increase with the increasing temperature. For example, the maximum solid solubilities of Co in Ni_13 − x_Co_x_In_9_ increased from 3.2 at.% Co at 673 K to 12.03 at.% Co at 873 K. However, the solid solubility range of Co_1−x_Ni_x_In_2_ was found to be 0–18.64% at. % Ni 673 K, while it shifted to the range of 3–20.1% at. % Ni at 873 K due to the absence of CoIn_2_ at 873 K.

### 3.3. Isothermal Sections of the Co-Ni-In Ternary System at 673 K and 873 K

**Figure 13:**
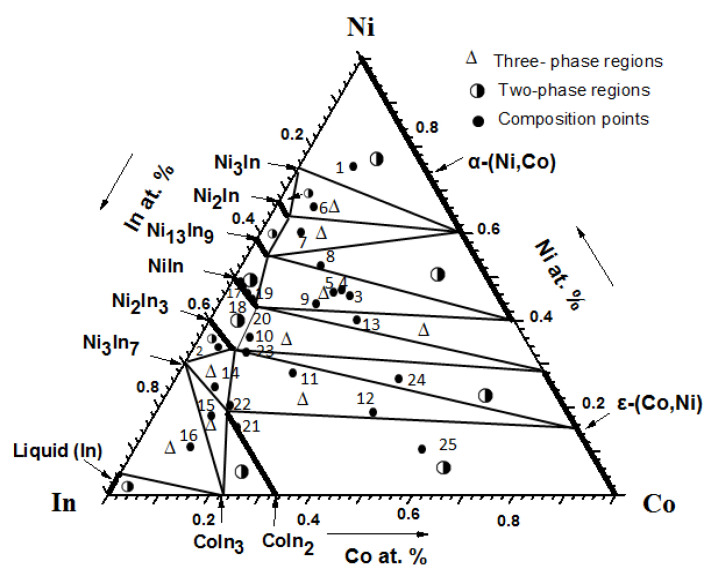
Isothermal section of the Co-Ni-In system at 673 K.

By comparing and analyzing more than 33 alloy samples of the Co-Ni-In ternary system and identifying the phases presented in each sample by XRD, optical microscopy, and SEM/EDS, the isothermal sections of the phase diagrams of the Co-Ni-In ternary system at 673 K and 873 K were determined. As shown in Figure 13, the isothermal section at 673 K consists of 11 single-phase regions, 21 two-phase regions, and 9 three-phase regions. The typical alloys and the details of the three-phase regions of the isothermal section of the Co-Ni-In ternary system are given in Table 2.

**Figure 14:**
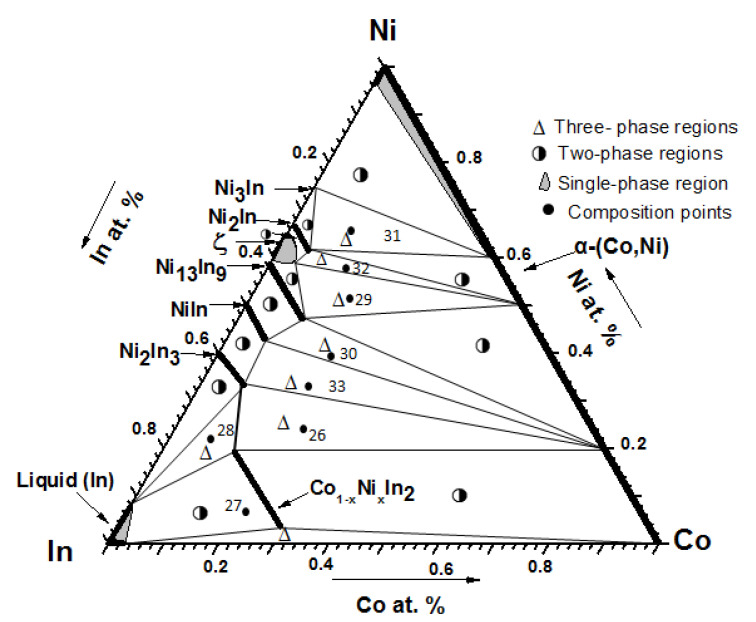
Isothermal section of the Co-Ni-In system at 873 K.

As shown in Figure 14, the isothermal section at 873 K contains 8 single-phase regions, 16 two-phase regions, and 8 three-phase regions. The typical alloys and the details of the three-phase regions of the Co-Ni-In isothermal section at 873 K are presented in Table 3.

By comparing the isothermal sections at 673 K and 873 K of phase diagram of the Co-Ni-In ternary system, the differences between the two sections can be found. The three three-phase regions, i.e., Co_1−x_Ni_x_In_2_ (x = 0.565) + Ni_2−x_Co_x_In_3_ (x = 0.4) + Ni_3_In_7_, Co_1−x_Ni_x_In_2_ (x = 0.565) + CoIn_3_ + Ni_3_In_7_ and CoIn_3_ + Ni_3_In_7_ + In (Liquid) disappear due to the absence of the binary compounds Ni_3_In_7_ and CoIn_3_ at 873 K [12,13], as seen Figure 4. The three-phase region Ni_13 − x_Co_x_In_9_ (x = 0.702) + Ni_1−x_Co_x_In_2_ (x = 0.091) + α-Ni_1−x_Co_x_ (x = 0.400) breaks into two three-phase regions, i.e., Ni_2−x_Co_x_In (x = 0.181) + ξ + α-Ni_1−x_Co_x_ (x = 0.500), and ξ + Ni_13 − x_Co_x_In_9_ (x = 2.634) + α-Ni_1−x_Co_x_ (x = 0.500) since the ξ phase exist from 746 K to 1223 K [13]. According to the Co-Ni binary phase diagram, the crystal structures of Co_1−x_Ni_x_ alloys belong to the hexagonal structure when the concentration of Ni is less than 10 at.% Ni at 673 K, while its structure starts to change from a hexagonal structure into a cubic structure when Ni content exceed 10 at.% Ni. However, the Co_1−x_Ni_x_ alloy only crystallizes in a cubic structure at 873 K. Therefore, the three-phase region Ni_1−x_Co_x_In (x = 0.125) + α-Co_1−x_Ni_x_ (x = 0.600) + ε-Co_1−x_Ni_x_ (x = 0.280) at 673 K becomes a two-phase region, Ni_1−x_Co_x_In (x = 0.160) + α-Ni_1−x_Co_x_ at 873 K. Although the binary compound Coln_2_ does not exist at 873 K, a narrow three-phase region, Co_1−x_Ni_x_In_2_ (x = 0.091) + α-Co + In (Liquid), presents at 873 K due to the solid solution of Co_1−x_Ni_x_ln_2_ (x = 0.091–0.612) appearing at 873 K.

### 3.4. Crystal Structure

#### 3.4.1. Crystal Structure of Ni_2−x_Co_x_In_3_ (x = 0.200)

**Figure 15:**
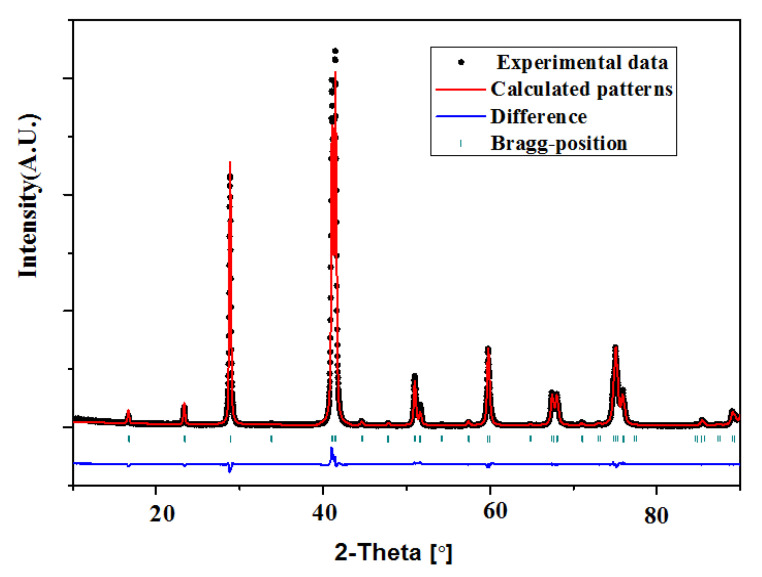
Observed, calculated, and differential XRD patterns of Ni_2−x_Co_x_In_3_ (x = 0.200).

The XRD and SEM/EDS data for the sample Ni_2−x_Co_x_In_3_ (x = 0.200) were collected in order to investigate its crystal structure. The XRD phase analysis points out that this sample is a single phase without any detectable impurity or additional phases. The SEM/EDS testing result shows that the composition of the sample is Ni34.62(3)Co5.23(4)In60.15(3), which reveals that 5.23(4)at.% of Co replaces the Ni position in Ni_2−x_Co_x_In_3_ (x = 0.200). To determine the crystal structures of the Ni_2−x_Co_x_In_3_ (x = 0.200) alloy, Rietveld refinement was performed from the XRD data by using the FullProf program. The powder X-ray diffraction pattern for the Ni_2−x_Co_x_In_3_ (x = 0.200) alloy is shown in Figure 15. The Rietveld refinement results of the alloys are listed in Table 4. The low values of the R_p_ and R_wp_ factors suggest that the fitted pattern is in good agreement with the experimental data and that the Rietveld refinement is reliable. The Rietveld refinement results support the case that the structure of sample remains unchanged at room temperature when cobalt is doped into the Ni_2−x_Co_x_In_3_ (x = 0.200) compound, and Ni_2−x_Co_x_In_3_ (x = 0.200) crystallizes in the Al_2_Ni_3_-type structure (space group P$\bar{3}$m1). The lattice parameters are a = 0.43959(5) nm, c = 0.53121(1) nm, and Z = 1. All positions are fully occupied in the compound. The Wyckoff 1a (0, 0, 0) site and 2d (1/3, 2/3, 0.3534 (3)) site are all occupied by In atoms, while the 2d (1/3, 2/3, 0.1381 (2)) site is occupied by Co and Ni atoms (0.1Co + 0.9 Ni).

#### 3.4.2. Crystal Structure of Co_1−x_Ni_x_In_2_ (x = 0.540, 0.580)

**Figure 16:**
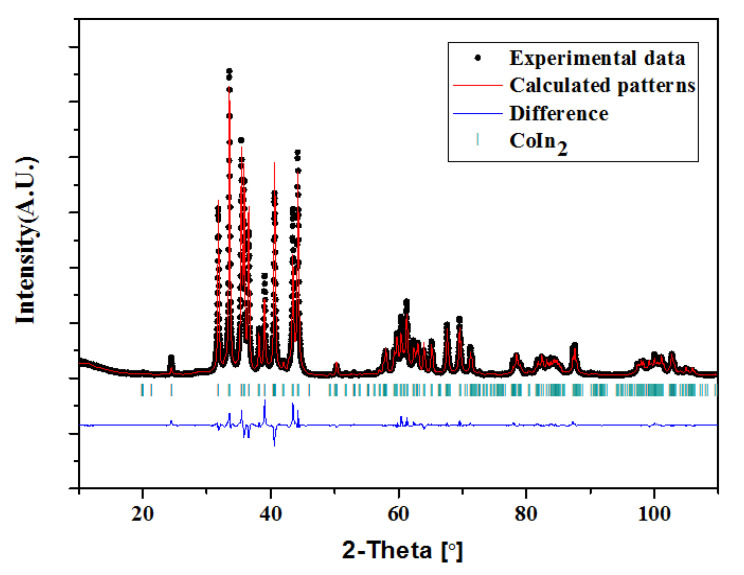
Observed, calculated and differential XRD patterns of Co_1−x_Ni_x_In_2_ (x = 0.540).

**Figure 17:**
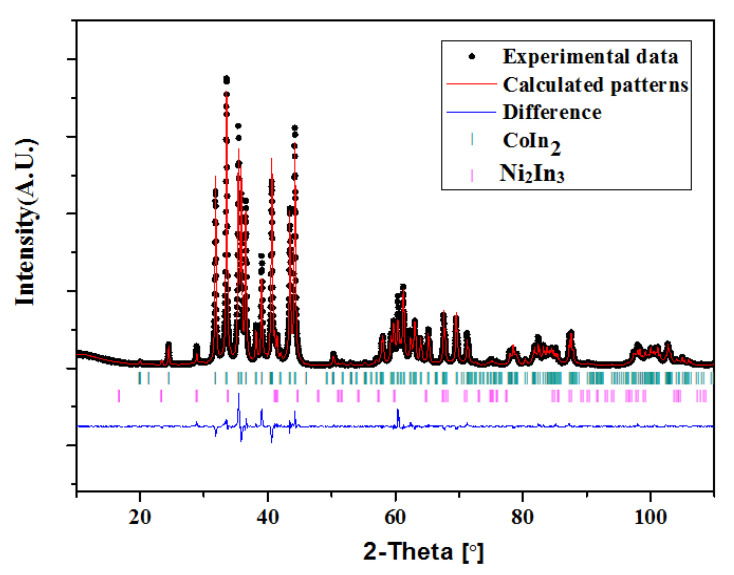
Observed, calculated and differential XRD patterns of Co_1−x_Ni_x_In_2_ (x = 0.580).

A large difference in the melting points of Co, Ni, and In (Co:1768K, Ni:1726K, In:429K) [37,38] causes difficulty in obtaining good single-phase Co_1−x_Ni_x_In_2_ samples. Thus, the crystal structure of Co_1−x_Ni_x_In_2_ was investigated with a few selected good single-phase samples. The crystal structure of Co_1−x_Ni_x_In_2_ (x = 0.540, 0.580) at 673 K was investigated via XRD and SEM/EDS. Figure 16 and Figure 17 present the XRD patterns of Co_1−x_Ni_x_In_2_. Table 4 lists the Rietveld refinement results of Co_1−x_Ni_x_In_2_. The lower values of R_p_ and R_wp_ for the Rietveld refinement indicate that the refinement results are credible. The SEM/EDS results show that the composition of Co_1−x_Ni_x_In_2_ (x = 0.580) is Co16.97(4)Ni18.02 (5)In65.01(5); see Table S1. The refinement result shows that the Co_1−x_Ni_x_In_2_ (x = 0.540) compound, in which a large number of Co atoms are replaced by Ni, remains in the single phase and maintains the Cu_2_Mg-type structure with the space group Fddd (No. 70). The lattice parameters of Co_1−x_Ni_x_In_2_ (x = 0.540) are refined to be a = 0.9424(3) nm, b = 0.5288(4) nm, and c = 1.7742(5) nm. The Co_1−x_Ni_x_In_2_ (x = 0.580) alloy contains the Co_1−x_Ni_x_In_2_ (x = 0.565) (Cu_2_Mg-type structure) phase with a small amount of the Ni_2−x_Co_x_In_3_ (x = 0.400) (Al_3_Ni_2_-type structure). The EDS results shows that the compositions of the Co_1−x_Ni_x_In_2_ (x = 0.565) and Ni_2−x_Co_x_In_3_ (x = 0.400) phases were about Co14.11(4)Ni18.64(3)In67.25(4) and Co7.41(3)Ni35.27(4)In57.3(4)2, which were identified as Co_1−x_Ni_x_In_2_ (x = 0.565) and Ni_2−x_Co_x_In_3_ (x = 0.400), respectively. This also proves that the solubility of Ni in Co_1−x_Ni_x_In_2_ is 18.64 at.% Ni in the Ni_2−x_Co_x_In_3_ (x = 0.400) alloy. The Rietveld refinement of the Co_1−x_Ni_x_In_2_ (x = 0.580) alloy shows that the mass fractions of these two phases in the sample were 2.7% and 97.3 %, respectively. The Rietveld refinement also indicated that the amount of Ni in the phase of Co_1−x_Ni_x_In_2_ was x = 0.565. This is in agreement with the results of solid solubility determination including the SEM/EDS measurements. The refined lattice parameters of Co_1−x_Ni_x_In_2_ (x = 0.565) are a = 0. 9421(2) nm, b = 0.5282(3) nm, and c = 1.7739(3) nm, which are slightly smaller than those of Co_1−x_Ni_x_In_2_ (x = 0.540). This phenomenon is mainly due to the similar crystal structure of Co and Ni, and the radius of Ni (R_Ni_ = 0.124 nm) atom is slightly smaller than that of Co (R_Co_ = 0.125 nm). The replacement of Ni atoms for Co atoms causes the volume of Co_1−x_Ni_x_In_2_ to shrink. This results in smaller sizes. The lattice parameters of Ni_2−x_Co_x_In_3_ (x = 0.400) are refined to be *a* = 0.4397(1) nm and 0.5319(3) nm. In the structure of Co_1−x_Ni_x_In_2_, the In atoms occupy the 16e and 16g sites, while Co atoms (including the substituting Ni atoms) exist at the 16g sites.

### 3.5. Magnetic Properties

#### 3.5.1. Magnetic Properties of Ni_2−x_Co_x_In_3_ (x = 0.200)

**Figure 18:**
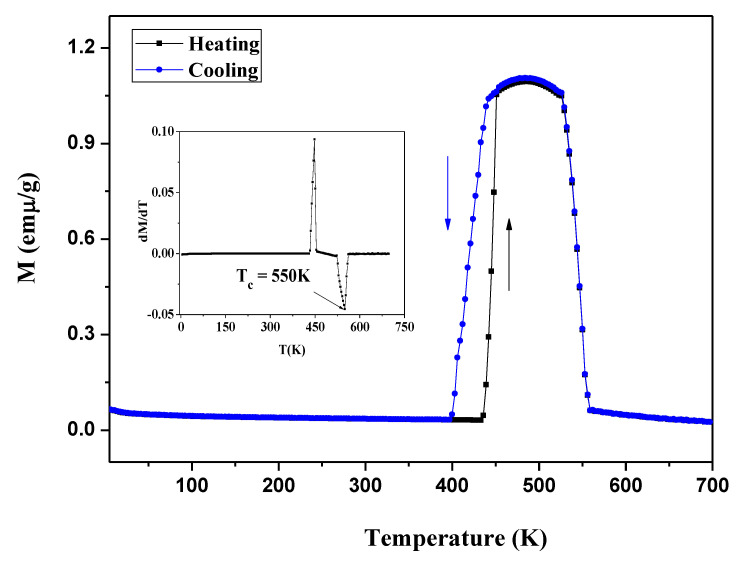
The zero-field-cooled (ZFC) and zero-field-heated (ZFH) temperature dependence of the magnetization under static magnetic fields of 50 mT for the Ni_2−x_Co_x_In_3_ (x = 0.200) alloy.

Figure 18 shows the zero-field-cooled (ZFC) and zero-field-heated (ZFH) temperature dependence of the magnetization under a static magnetic field of 50 mT for the Ni_2−x_Co_x_In_3_ (x = 0.200) alloy. During the heating process, the magnetization of the alloy remains constant at 0.044 emµ/g in the range of 5–436 K, and then increases sharply at 436 K (T_HS_) and rises to 1.05 emμ/g at 451 K (T_HF_). After that, the magnetization increases slowly and reaches a maximum of 1.11 emμ/g at 482 K. When the temperature increases from 482 to 528 K, the magnetization slowly drops to 1.05 emµ/g. The magnetization drops rapidly to a minimum value of 0.046 emµ/g with a further temperature increase to 560 K. The cooling curve almost completely coincides with the heating one, except in the temperature range of 339–451 K. In the cooling curve, the magnetization starts to decrease at 446 K (T_CS_) and stops to decrease at 399 K (T_CF_). The temperature cooling curve shifts to the low-temperature direction, indicating that the sample has thermal hysteresis in this temperature range; Δ = 21 K (ΔT_hys_= (T_HS_ + T_HF_ – T_CS_ − T_CF_)/2). This is very similar to the martensitic transformation phenomenon occurring in the Ni-Mn-A (A = Ga, Sn, In) [39,40,41] systems. As can be seen from the inset of Figure 18, the differential of magnetization to temperature varies with temperature and shows that the Curie temperature of the compound is 550 K.

**Figure 19:**
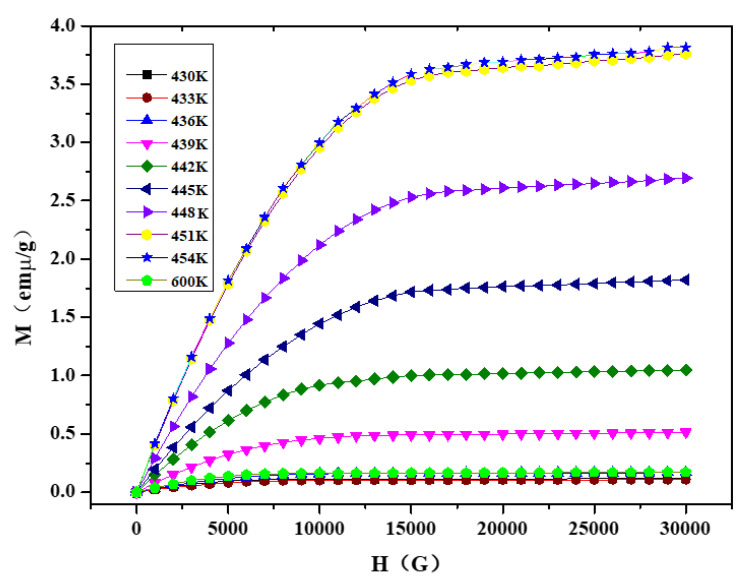
Magnetization isotherms of the Ni_2−x_Co_x_In_3_ (x = 0.200) alloy.

Magnetization isotherms, measured during heating cycles at different temperatures, are shown in Figure 19. The samples show ferromagnetism at all measuring temperatures. Clearly, the magnetization increases with the increase in temperature. The magnetization increases from 0.178 emμ/g to 3.84 emμ/g when temperature is heated from 430 K to 454 K under the 3 T magnetic field. The sample has weak magnetism at 600 K—the magnetization is only 0.147 at 3 T. Magnetic entropy change (ΔS_M_) in the system, due to the application of external magnetic field, can be determined from Maxwell’s relation (Equation (1)) by integrating the magnetization isotherms over the magnetic field.

(1) $$\Delta S_{M}{(T,H}_{\max}{)=S}_{M}{(T,H}_{\max}{)-S}_{M}\left(T,0\right)=\int_{0}^{H_{\max}}{\left(\frac{\partial M}{\partial H}\right)}_{H}\mathrm{dH}$$

**Figure 20:**
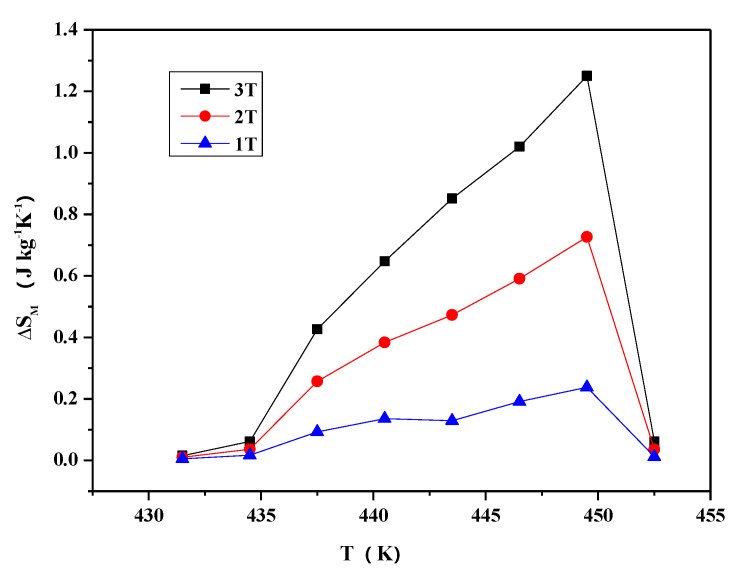
The magnetic entropy changes as a function of temperature under 3 T, 2 T, 1 T magnetic fields for the Ni_2−x_Co_x_In_3_ (x = 0.200) alloy.

Magnetic entropy changes in the alloy have been derived from the isothermal magnetization curves. Figure 20 shows the magnetic entropy change in the Ni_2−x_Co_x_In_3_ (x = 0.200) sample as a function of temperature near 445 K under the 3 T, 2 T, and 1 T magnetic fields. Clearly, the greater the magnetic entropy, the stronger the magnetic field. The peak value under a field change of 3 T is 1.25 J kg^−1^K^−1^ at T = 449.5 K. The relative cooling power (R_CP_ = ΔS_M_ × ΔT_FWHM_), which is the measure of the amount of heat transfer between the cold and hot reservoirs in an ideal refrigeration cycle, is evaluated to be 14.125 J kg^−1^K^−1^ in the vicinity of 430–454 K for 3 T magnetic field change, where ΔT_FWHM_ = 11.3 K.

#### 3.5.2. Magnetic Properties of Co_1−x_Ni_x_In_2_ (x = 0.540)

**Figure 21:**
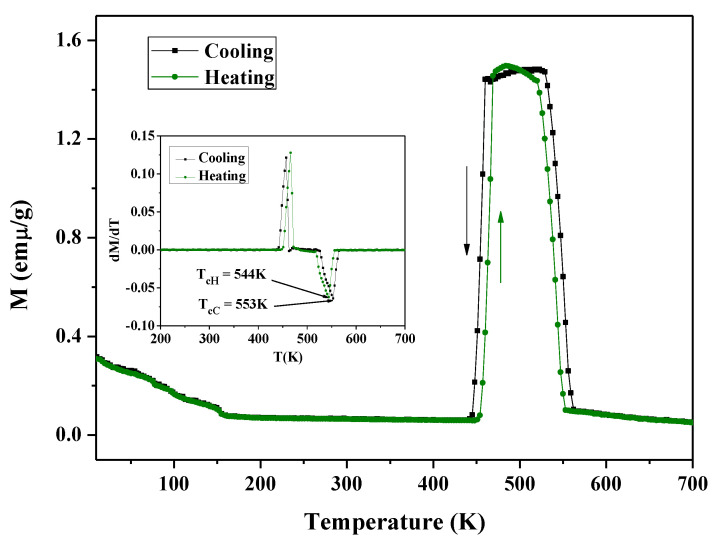
The ZFC and ZFH temperature dependence of the magnetization under static magnetic fields of 50 mT for the Co_1−x_Ni_x_In_2_ (x = 0.540) compound.

**Figure 22:**
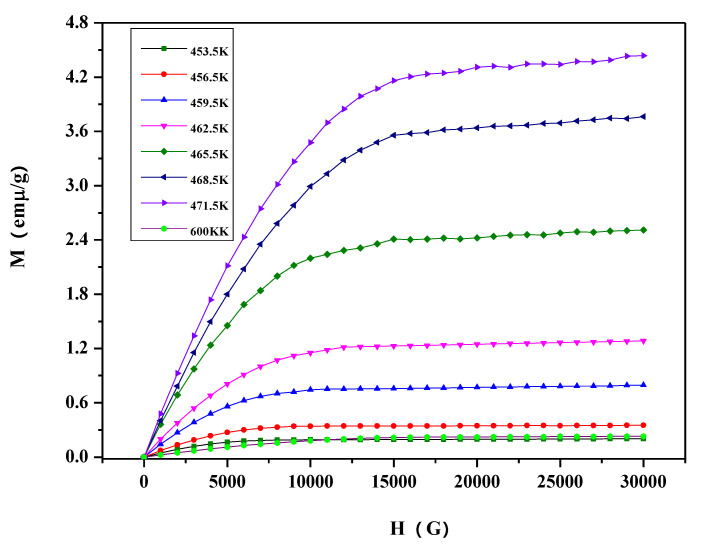
Magnetization isotherms for the Co_1−x_Ni_x_In_2_ (x = 0.540) alloy.

**Figure 23:**
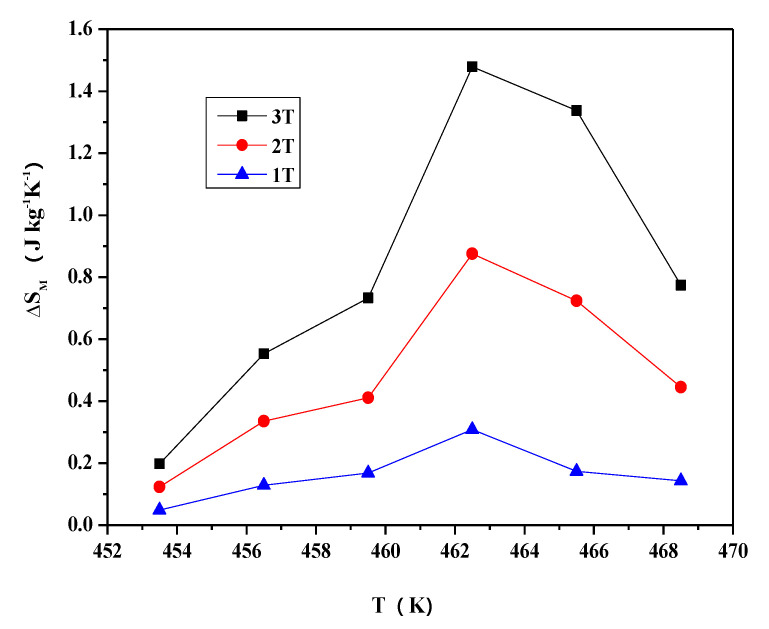
The magnetic entropy changes as a function of temperature under 3 T, 2 T, and 1 T magnetic fields for the Co_1−x_Ni_x_In_2_ (x = 0.540) alloy.

Figure 21 shows the magnetization behavior of the Co_1−x_Ni_x_In_2_ (x = 0.540) alloy during thermal cycling with a heating/cooling rate of 5 K/min at ZFC and ZFH. The tendency of magnetization changes with temperature of the Co_1−x_Ni_x_In_2_ (x = 0.540) compound is very similar to that of Ni_2−x_Co_x_In_3_ (x = 0.200). However, there are two hysteresis taking place in the Co_1−x_Ni_x_In_2_ (x = 0.540) alloy. The ΔT_hys_ in low temperature is 8.5 K, which is smaller than Ni_2 – x_Co_x_In_3_ (x = 0.200). As can be seen from the inset of Figure 21, due to the thermal hysteresis, two different Curie temperatures (544 K and 553 K) can be obtained from the heating curve and the cooling curve. Representative isothermal magnetization loops measured around 462.5 K for Co_1 – x_Ni_x_In_2_ (x = 0.540) are presented in Figure 22. The sample shows a low magnetization below 459 K, which rises from 0.21 emµ/g to 4.46 emµ/g with a temperature increase from 453 K to 471 K at the magnetic field of 3T. Associated with this magnetostructural transition, a large magnetic entropy change occurs. Figure 23 shows the magnetic entropy change as a function of temperature under the 3 T, 2 T, and 1 T magnetic fields for the alloy. It is clearly seen that as the magnetic field increases, the magnetic entropy of the Co_1−x_Ni_x_In_2_ (x = 0.540) compound becomes larger. Under a magnetic field of 3 T, the magnetic entropy of the alloy reaches a maximum of 1.475 J kg^−1^K^−1^at about 463.5 K. The corresponding half-maximum width is ΔT_FWHM_ = 8.9 K for the compound. The direct R_CP_ is evaluated as 13.128 J kg^−1^K^−1^.

**Figure 24:**
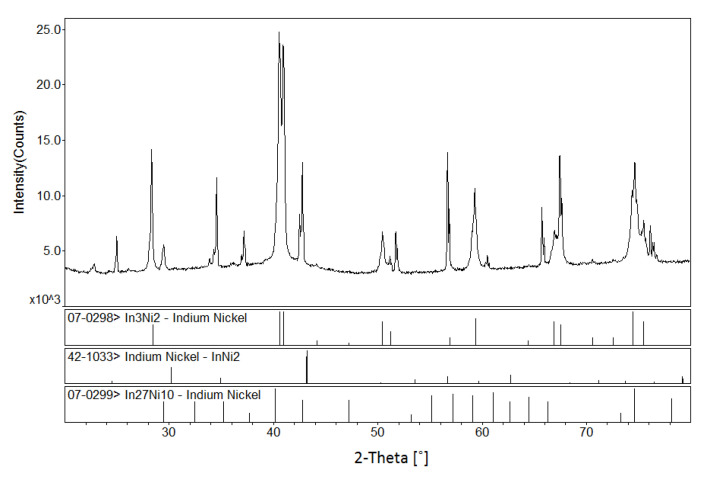
The XRD patterns of sample Ni_2−x_Co_x_In_3_ (x = 0.200) at 490 K.

**Figure 25:**
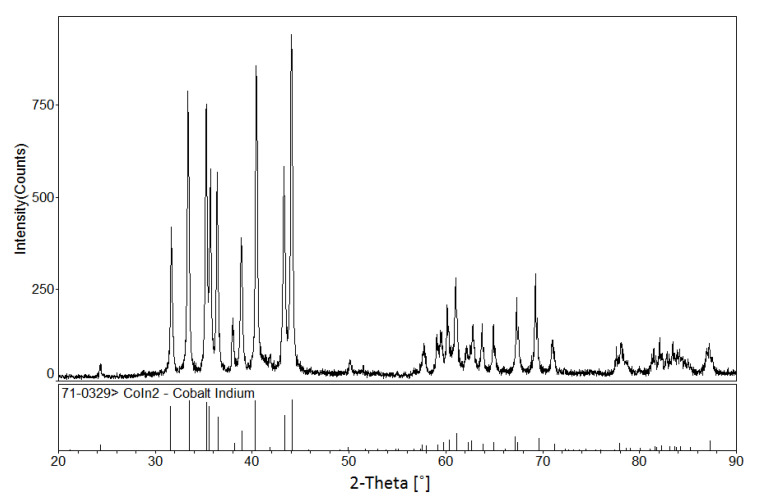
The XRD patterns of sample Co_1−x_Ni_x_In_2_ (x = 0.540) at 490 K.

Since the magnetization dependence of temperature (MT) curves of Ni_2−x_Co_x_In_3_ (x = 0.200) and Co_1−x_Ni_x_In_2_ (x = 0.540) are similar to those of ferromagnetic shape memory alloys with martensitic transformation, temperature-dependent powder XRD measurements were performed in order to obtain further insights into whether the compound undergoes martensite transformation behavior. Figure 24 shows the XRD patterns for the Ni_2−x_Co_x_In_3_ (x = 0.200) alloy measured at 490 K. According to XRD patterns, the main phase of the alloy at 490 K still was a Ni_2_In_3_ phase with an Al_2_Ni_3_-type structure. Furthermore, a small amount of Ni_10_In_27_ (space group Im-3 m) and Ni_2_In (space group P63/mmc) appears in alloy. The results indicate that a small amount of Ni_10_In_27_ and Ni_2_In is generated during heating. Indexing of the Ni_2_In_3_ phase showed that a = 0.43905 (5) nm, c = 0.52930 (3) nm, and V = 0.08836 nm^3^. All these results show that a structure phase transformation but not a martensitic transformation occurred in Ni_2−x_Co_x_In_3_ (x = 0.200) during heating. Figure 25 shows the XRD patterns of the Co_1−x_Ni_x_In_2_ (x = 0.540) alloy at 490 K, which still retains the Cu_2_Mg-type structure type at 490 K. These results point to the notion that no martensitic transformation occurred in the Co_1−x_Ni_x_In_2_ (x = 0.540) alloys during heating.

## 4. Conclusions

More than 130 samples were prepared and investigated by experimental methods to establish the phase equilibrium of the ternary Co-Ni-In system at 673 K and 873 K. The peculiar MT curves of Ni_2−x_Co_x_In_3_ (x = 0.200) and Co_1−x_Ni_x_In_2_ (x = 0.540) allow them to have potential to become magnetic functional materials.

1. There are eight Co-Ni-In binary compounds existing at 673 K and six binary compounds existing at 873 K. Compared to the phases at 673 K, the binary compounds Ni_3_In_7_, CoIn_3_, and CoIn_2_ disappeared, and ξ exists at 873 K.
2. The solid solubility range of Co_1−x_Ni_x_In_2_ was found to be 0–18.64% at. % Ni at 673 K, while it shifted to the range of 3–20.1% at. % Ni at 873 K due to the absence of CoIn_2_ at 873 K.
3. The isothermal section of the phase diagram of the Co-Ni-In system at 673 K consists of 11 single-phase regions, 21 two-phase regions, and 9 three-phase regions, and the isothermal section at 873 K contains 8 single-phase regions, 16 two-phase regions, and 8 three-phase regions. Three-phase regions disappear due to the absence of the binary compounds Ni_3_In_7_ and CoIn_3_ at 873 K. The three-phase region Ni_13 − x_Co_x_In_9_ (x = 0.702) + Ni_1−x_Co_x_In_2_ (x = 0.091) + α-Ni_1−x_Co_x_ (x = 0.400) breaks into two three-phase regions due to the ξ phase existing at 873 K.
4. The MT curves of Ni_2−x_Co_x_In_3_ (x = 0.200) and Co_1−x_Ni_x_In_2_ (x = 0.540), which are similar to the Ni-Mn-A (A = Ga, Sn, In), do not undergo martensitic transformation at temperatures, which results in a sharp change in magnetization. The maximum magnetic entropy changes of Ni_2−x_Co_x_In_3_ (x = 0.200) and Co_1−x_Ni_x_In_2_ (x = 0.540) under 3T in 430 K–454 K and 454 K–472 K are 1.25 J kg^−1^K^−1^, 1.475 J kg^−1^K^−1^, respectively.

## Figures and Tables

**Table 1:** Crystallographic data of the compounds in the Co-Ni-In system.

Compounds	Structure Type	Space Group	Lattice Parameters (nm)				References
			a	b	c	β(°)	
CoIn_2_	CuMg_2_	Fddd	0.9402	0.5282	1.7846	90	[30]
CoIn_3_	Mg	P6_3_/mmc	0.6829	0.6829	0.7094	90	[30]
Ni_4_In	W	Im$\bar{3}$m	0.2929	0.2929	0.2929	90	[33]
Ni_3_In	Ni_3_Sn	P6_3_/mmc	0.5320	0.5320	0.4242	90	[31]
Ni_3_In	AuCu_3_	Pm$\bar{3}$m	0.3750	0.3750	0.3750	90	[31]
ζ	NiAs	P6_3_/mmc	0.4189	0.4189	0.5123	90	[31]
Ni_2_In	Ni_2_In	P6_3_/mmc	0.4179	0.4179	0.5131	90	[31]
Ni_13_In_9_	Ni_13_In_9_	C2/m	1.4646	0.8329	0.8977	35.35	[31]
Ni_13_In_7_	AsNi	P6_3_/mmc	0.4178	0.4178	0.5137	90	[33]
NiIn	CoSn	P6/mmm	0.5243	0.5243	0.4342	90	[31]
NiIn	CsCl	Pm$\bar{3}$m	0.3092	0.3092	0.3092	90	[31]
Ni_2_In_3_	Al_3_Ni_2_	P$\bar{3}$m1	0.4390	0.4390	0.5201	90	[31]
Ni_3_In_7_	Cu_5_Zn_8_	I4$\bar{3}$m	0.9180	0.9180	0.9180	90	[33]
Ni_2_CoIn	BiF_3_	Fm$\bar{3}$m	0. 5944	0. 5944	0. 5944	90	[32]

**Table 2:** Typical samples and details of three-phase regions in the Co-Ni-In ternary system at 673 K.

Phase Regions	Samples	Alloy Compositions	Phase Components
1	No. 6	Co10Ni68In22	Ni_2−x_Co_x_In(x = 0.091) + Ni_3_In+ α-Ni_1−x_Co_x_ (x = 0.400)
2	No. 7	Co10Ni62In28	Ni_13 − x_Co_x_In_9_ (x = 0.702) + Ni_2−x_Co_x_In (x = 0.091) + α-Ni_1−x_Co_x_ (x = 0.400)
3	No. 9	Co20Ni45In35	Ni_13 − x_Co_x_In_9_ (x = 0.702) + Ni_1−x_Co_x_In (x = 0.125) + α-Co_1−x_Ni_x_(x = 0.600)
4	No. 10	Co10Ni40In50	Ni_1−x_Co_x_In (x = 0.125) + Ni_2−x_Co_x_In_3_ (x = 0.400) + ε-Co_1−x_Ni_x_ (x = 0.280)
5	No. 11	Co40Ni20In40	Co_1−x_Ni_x_In_2_ (x = 0.565) + Ni_2−x_Co_x_In_3_ (x = 0.400) + ε-Co_1−x_Ni_x_ (x = 0.280)
6	No. 13	Co30Ni40In30	Ni_1−x_Co_x_In (x = 0.125) + α-Co_1−x_Ni_x_ (x = 0.600) +ε-Co_1−x_Ni_x_ (x = 0.280)
7	No. 14	Co10Ni24In66	Co_1−x_Ni_x_In_2_ (x = 0.565) + Ni_2−x_Co_x_In_3_ (x = 0.400) + Ni_3_In_7_
8	No. 15	Co16Ni12In72	Co_1−x_Ni_x_In_2_ (x = 0.565) + CoIn_3_ + Ni_3_In_7_
9	No. 16	Co10Ni12In78	CoIn_3_ + Ni_3_In_7_ + In (Liquid)

**Table 3:** Typical samples and details of three-phase regions in the Co-Ni-In ternary system at 873 K.

Phase Regions	Samples	Alloy Compositions	Phase Components
I	No. 26	Co28Ni24In48	Co_1−x_Ni_x_In_2_ (x = 0.612) + Ni_2−x_Co_x_In_3_ (x = 0.450) + α-Co_1−x_Ni_x_ (x = 0.200)
II	No. 28	Co8Ni22In70	Co_1−x_Ni_x_In_2_ (x = 0.612) + Ni_2−x_Co_x_In_3_ (x = 0.450) + In (Liquid)
III	No. 28	Co18Ni52In30	ξ + Ni_13 − x_Co_x_In_9_ (x = 2.634) + α-Ni_1−x_Co_x_ (x = 0.500)
IV	No. 30	Co20Ni45In35	Ni_13 − x_Co_x_In_9_ (x = 2.634)+ Ni_1−x_Co_x_In (x = 0.160) + α-Ni_1−x_Co_x_ (x = 0.600)
V	No. 31	Co14Ni66In20	Ni_3_In + Ni_2−x_Co_x_In (x = 0.181) + α-Ni_1−x_Co_x_ (x = 0.400)
VI	No. 32	Co12Ni58In30	Ni_2−x_Co_x_In (x = 0.181) + ξ + α-Ni_1−x_Co_x_ (x = 0.500)
VII	No. 33	Co20Ni34In46	Ni_1−x_Co_x_In (x = 0.160) + Ni_2−x_Co_x_In_3_ (x = 0.450) + α-Co_1−x_Ni_x_ (x = 0.200)
VIII			Co_1−x_Ni_x_In_2_ (x = 0.091) + α-Co + In (Liquid)

**Table 4:** Crystallographic data and Rietveld refinement results for Ni_2−x_Co_x_In_3_ (x = 0.200) and Co_1−x_Ni_x_In_2_ (x= 0.54, 0.58) alloys.

Compound	Phase	Structure Type	Space Group	Lattice Parameters (nm)			Atomic Parameters			Reliability Factors		Goodness of Fit
				a	b	c	Atoms	Wyck (x, y, z)	Occ	R _p_(%)	R _exp_(%)	S (%)
Ni_2−x_Co_x_In_3_(x = 0.200)	Ni_2−x_Co_x_In_3_ (x = 0.200)	Al_2_Ni_3_	P$\bar{3}$m1 (164)	0.43959(5)	0.43959(5)	0.53121(1)	In1	1a (0,0,0)	1	10.46	14.15	3.2
							In2	2d (1/3, 2/3, 0.6469(3))	1			
							M1 = (Co, Ni)	2d (0, 0, 0.1381(2))	0.1Co + 0.9 Ni			
Co_1−x_Ni_x_In_2_(x = 0.540)	Co_1−x_Ni_x_In_2_(x = 0.540)	CuMg_2_	Fddd (70)	0.9424(3)	0.5288(4)	1.7742(5)	In1	16e (0.9648(2), 1/4,1/4)	1	10.29	13.26	2.9
							M2 = (Co, Ni)	16g (1/4, 0.9963(7), 1/4)	0.466Co + 0.534Ni			
							In2	16g (1/4, 0.7120(5), 1/4)	1			
Co_1−x_Ni_x_In_2_ (x = 0.580)	Co_1−x_Ni_x_In_2_(x = 0.565)	CuMg_2_	Fddd (70)	0.9421(2)	0.5282(3)	1.7739(3)	In1	16e (0.9642(3), 1/4,1/4)	1	9.89	12.58	2.7
							M3 = (Co, Ni)	16g (1/4, 0.9962(2), 1/4)	0.438Co + 0.562Ni			
							In2	16g (1/4, 0.7131(4), 1/4)	1			
	Ni_2−x_Co_x_In_3_ (x = 0.400)	Al_3_Ni_2_	P$\bar{3}$m1 (164)	0.4397(1)	0.4397(1)	0.5319(3)	In1	1a (0 0 0)	1	9.89	12.58	2.7
							In2	2d (1/3, 2/3, 0.6412(2))	1			
							M4 = (Co, Ni)	2d (0, 0, 0.1350)	0.8Co + 0.2Ni			

M1 = 0.1Co + 0.9 Ni; M2 = 0.466Co + 0.534Ni; M3 = 0.438Co + 0.562Ni; M4 = 0.8Co + 0.2Ni

## Supplementary Materials

The following are available online at https://www.mdpi.com/1996-1944/13/18/3990/s1, Table S1: XRD and SEM/EDS analysis results of the selected Co-Ni-In samples at 673 K; Table S2: XRD and SEM/EDS analysis results of the selected Co-Ni-In samples at 873 K.
