# Supplementary material for: The Phase Relations of the Co-Ni-In Ternary System at 673 K and 873 K and Magnetic Properties of Their Compounds
Source: Materials (Basel). 2020 Sep 9;13(18):3990. doi: 10.3390/ma13183990 (PMC7558481; doi:10.3390/ma13183990)
Supplement: Supplementary file 1 [file materials-13-03990-s001.pdf]

Article

The Phase Relations of the Co-Ni-In Ternary System at 673 K and 873 K and Magnetic Properties of Their Compounds

Tonghan Yang [1,2], Wei He [1,2,*], Guojian Chen [2], Weijing Zeng [2], Jinzhi Wang [3], Lingmin Zeng [2] and Jianlie Liang [4]

[1] College of Chemistry and Chemical Engineering, Guangxi University, Nanning 530004, China; yangthan199@163.com
[2] School of Resources, Environment and Materials and Guangxi Key Laboratory of Processing for Non-ferrous Metallic and Featured Materials, Guangxi University, Nanning 530004, China; chgjxx@163.com (G.C.); zmszengweijing@163.com (W.Z.); lmzeng@gxu.edu.cn (L.Z.)
[3] School of Materials and Chemical Engineering, Ningbo University of Technology, Ningbo 315211, China; wangjz@nbut.edu.cn
[4] School of Science, Guangxi University of Nationalities, Nanning, 530006, China; liangjl1971@126.com
* Correspondence: wei_he@gxu.edu.cn; Tel.: +86-771-327-5918 or +86-771-323-9406

Received: 10 July 2020; Accepted: 4 September 2020; Published: date

Table S1. XRD and SEM/EDS analysis results of the selected Co-Ni-In samples at 673 K.

Sample No.	Nominal composition (at. %)			Phase	XRD analysis				Phase composition (at.%) measured by EDS		
	Co	Ni	In		space group	Lattice constant(nm)					
						a	b	c	Co	Ni	In
1	10	75	15	α-$Ni_{1-x}Co_x$ (x = 0.355)	$Fm\bar{3}m$(225)	0.3516(4)			35.53(5)	62.15(4)	2.31(4)
				Ni_3In	$P6_3/mmc$(194)	0.5542(2)		0.4226(3)	1.02(5)	72.26(4)	26.72(5)
2	4	34	62	$Ni_{2-x}Co_xIn_3$ (x = 0.118)	$P\bar{3}m1$(164)	0.4397(2)		0.5319(3)	2.36(4)	59.12(3)	38.52(3)
				Ni_3In_7	$Im\bar{3}m$(229)	0.9178(3)			0.86(6)	32.43(6)	66.72(5)
3	25	50	25	$Ni_{13-x}Co_xIn_9$ (x = 0.702)	C2/m(12)	1.4654(6)	0.8342(5)	0.8968(5)	1.02(4)	58.14(5)	40.84(5)
				$Ni_{1-x}Co_xIn$ (x = 0.125)	P6/mmm (191)	0.52426(8)		0.43474(6)	3.37(6)	46.96(6)	49.67(6)

				α-$Ni_{1-x}Co_x$ (x = 0.600)	$Fm\bar{3}m$(225)	0.3519(3)			61.12(5)	38.28(6)	0.60(5)
4	22	51	27	$Ni_{13-x}Co_xIn_9$ (x = 0.702)	C2/m(12)	1.4652(1)	0.8343(7)	0.8968(3)	1.43(6)	57.66(6)	41.24(6)
				$Ni_{1-x}Co_xIn$ (x = 0.125)	P6/mmm (191)	0.52421(5)		0.43474(1)	2.72(7)	47.80(7)	49.48(7)
				α-$Ni_{1-x}Co_x$ (x = 0.600)	$Fm\bar{3}m$(225)	0.3519(5)			60.52(7)	39.12(7)	0.36(7)
5	27	48	52	$Ni_{13-x}Co_xIn_9$ (x = 0.702)	C2/m(12)	1.4653(3)	0.8345(2)	0.8967(2)			
				$Ni_{1-x}Co_xIn$ (x = 0.125)	P6/mmm (191)	0.52421(7)		0.43472(4)			
				α-$Ni_{1-x}Co_x$ (x = 0.600)	$Fm\bar{3}m$(225)	0.3518(3)					
6	10	68	22	$NiIn_3$	$P6_3$/mmc(194)	0.5543(3)		0.4227(5)			
				$Ni_{2-x}Co_xIn$ (x = 0.091)	$P6_3$/mmc (194)	0.4187(1)		0.5145(3)			
				α-$Ni_{1-x}Co_x$ (x=0.400)	$Fm\bar{3}m$(225)	0.3516(4)					
7	10	62	28	$Ni_{2-x}Co_xIn$ (x = 0.091)	$P6_3$/mmc (194)	0.4185(3)		0.5145(3)	2.05(5)	63.82(4)	34.14(4)
				$Ni_{13-x}Co_xIn_9$ (x = 0.702)	C2/m(12)	1.4654(2)	0.8344(4)	0.8966(2)	2.7(4)	57.73(5)	39.57(7)
				α-$Ni_{1-x}Co_x$ (x=0.400)	$Fm\bar{3}m$(225)	0.3513(6)			33.98(5)	65.13(5)	0.89(5)
8	20	55	25	$Ni_{13-x}Co_xIn_9$ (x = 0.702)	C2/m(12)	1.4655(5)	0.8342(6)	0.8968(3)	2.98(3)	57.44(4)	39.58(5)
				α-$Ni_{1-x}Co_x$ (x=0.400)	$Fm\bar{3}m$(225)	0.3516(4)			33.62(6)	66.01(5)	0.37(5)
9	20	45	35	$Ni_{13-x}Co_xIn_9$ (x = 0.702)	C2/m(12)	1.4654(4)	0.8342(5)	0.8968(2)	1.62(5)	57.13(4)	41.24(5)
				$Ni_{1-x}Co_xIn$ (x = 0.125)	P6/mmm (191)	0.5242 (2)		0.43471(8)	3.02(4)	47.56(5)	49.42(5)

				α-$Ni_{1-x}Co_x$ (x = 0.600)	$Fm\bar{3}m$(225)	0.3516(4)			60.52(5)	39.12(6)	0.36(4)
10	10	40	50	$Ni_{1-x}Co_xIn$ (x = 0.125)	P6/mmm (191)	0.52423(6)		0.43472(6)	5.82(5)	44.89(5)	49.29(7)
				$Ni_{2-x}Co_xIn_3$ (x = 0.400)	$P\bar{3}m1$(164)	0.4398(1)		0.5317(3)	7.99(4)	33.02(5)	58.99(5)
				ε-$Co_{1-x}Ni_x$ (x = 0.280)	$P6_3$/mm(194)	0.2505(1)		0.4061(2)	72.31(5)	27.03(5)	0.66(6)
11	29.8	25.5	44.7	$Ni_{2-x}Co_xIn_3$ (x = 0.400)	$P\bar{3}m1$(164)	0.43976(5)		0.53192(6)	4.38(5)	36.54(5)	59.07(5)
				$Co_{1-x}Ni_xIn_2$ (x=0.565)	Fddd(70)	0.94216(5)	0.5281(3)	1.7738(2)	18.64(4)	16.12(5)	65.25(5)
				ε-$Co_{1-x}Ni_x$ (x = 0.280)	$P6_3$/mm(194)	0.2502(1)		0.4063(2)	83.58(5)	15.56(4)	0.87(5)
12	40	20	40	$Ni_{2-x}Co_xIn_3$ (x = 0.400)	$P\bar{3}m1$(164)	0.43978(7)		0.52935(5)	7.68(6)	33.45(6)	58.87(7)
				$Co_{1-x}Ni_xIn_2$ (x=0.565)	Fddd(70)	0.9422(2)	0.5281(3)	1.7738(3)	15.67(6)	17.94(7)	66.39(8)
				ε-$Co_{1-x}Ni_x$ (x = 0.280)	$P6_3$/mm(194)	0.2504(5)		0.40612(7)	88.27(5)	11.23(5)	0.5(5)
13	30	40	30	$Ni_{1-x}Co_xIn$ (x = 0.125)	P6/mmm (191)	0.5243(1)		0.43471(6)	4.89(5)	47.12(6)	49.99(4)
				α-$Co_{1-x}Ni_x$ (x = 0.600)	$Fm\bar{3}m$(225)	0.3519(5)			58.92(5)	39.43(5)	1.65(4)
				ε-$Co_{1-x}Ni_x$ (x = 0.280)	$P6_3$/mm(194)	0.2503(3)		0.4061(4)	75.02(6)	23.61(5)	1.37(5)
14	10	24	66	$Co_{1-x}Ni_xIn_2$ (x = 0.565)	Fddd(70)	0.9421(5)	0.5282(5)	1.7739(4)			
				$Ni_{2-x}Co_xIn_3$ (x = 0.400)	$P\bar{3}m1$(164)	0.4396(1)		0.5318(2)			
				Ni_3In_7	$Im\bar{3}m$(229)	0.9177(2)					
15	16	12	72	$Co_{1-x}Ni_xIn_2$ (x = 0.565)	Fddd(70)	0.9421(1)	0.5282(3)	1.7739(3)			
				$CoIn_3$	$P\bar{4}n2$	0.6817(3)		0.7088(2)			

				Ni_3In_7	$Im\bar{3}m(229)$	0.9178(3)					
				In (Liquid)							
16	10	12	78	$CoIn_3$	$P\bar{4}n2$	0.6817(5)		0.7088(4)			
				Ni_3In_7	$Im\bar{3}m(229)$	0.9178(6)					
				In (Liquid)							
17	2	48	50	$Ni_{1-x}Co_xIn$ (x = 0.040)	P6/mmm (191)	0.52378(5)		0.43442(5)	2.31(3)	47.97(3)	49.72(4)
18	4	46	50	$Ni_{1-x}Co_xIn$ (x = 0.080)	P6/mmm (191)	0.52387(4)		0.43451(4)	3.89(2)	45.79(3)	50.32(3)
19	6	44	50	$Ni_{1-x}Co_xIn$ (x = 0.120)	P6/mmm (191)	0.52398(3)		0.43458(3)	6.22(4)	44.75(4)	19.03(5)
20	7	43	50	$Ni_{1-x}Co_xIn$ (x = 0.125)	P6/mmm (191)	0.52405(3)		0.43465(3)	5.75(5)	45.02(6)	49.23(6)
				$Ni_{2-x}Co_xIn_3$ (x = 0.400)	$P\bar{3}m1(164)$	0.4397(1)		0.5319(3)	7.82(4)	33.11(5)	58.07(5)
				ε-$Co_{1-x}Ni_x$ (x = 0.280)	$P6_3$/mm(194)	0.2503(1)		0.4061(2)	72.81(7)	26.18(6)	1.01(5)
21	16	18	66	$Co_{1-x}Ni_xIn_2$ (x = 0.540)	Fddd(70)	0.9424(3)	0.5288(4)	1.7742(5)	16.97(4)	18.02(5)	65.01(5)
22	14	20	66	$Co_{1-x}Ni_xIn_2$ (x = 0.560)	Fddd(70)	0.9421(2)	0.5282(3)	1.7739(3)	14.11(4)	18.64(3)	67.25(4)
				$Ni_{2-x}Co_xIn_3$ (x = 0.380)	$P\bar{3}m1(164)$	0.4397(1)		0.5319(3)	7.41(3)	35.27(4)	57.32(4)
23	10	30	60	$Ni_{2-x}Co_xIn_3$ (x = 0.160)	$P\bar{3}m1(164)$	0.4397(1)		0.5319(3)	3.28(6)	37.18(5)	59.54(7)
				ε-$Co_{1-x}Ni_x$ (x = 0.140)	$P6_3$/mm(194)	0.2503(1)		0.4061(2)	85.11(7)	13.61(8)	1.28(8)
24	50	25	25	$Ni_{2-x}Co_xIn_3$ (x = 0.189)	$P\bar{3}m1(164)$	0.4397(1)		0.5319(3)	3.78(6)	36.59(6)	59.63(7)
				ε-$Co_{1-x}Ni_x$ (x = 0.140)	$P6_3$/mm(194)	0.2503(1)		0.4061(2)	85.32(5)	14.02(5)	0.66(5)

25	50	10	40	$Co_{1-x}Ni_xIn_2$ (x = 0.477)	Fddd(70)	0.9421(2)	0.5282(3)	1.7739(3)	17.62(5)	15.77(6)	66.61(4)
				ε-$Co_{1-x}Ni_x$ (x = 0.124)	$P6_3/mm(194)$	0.2503(1)		0.4061(2)	86.03(5)	12.41(7)	1.56(6)

Table S2. XRD and SEM/EDS analysis results of the selected Co-Ni-In samples at 873 K.

**Sample No.**	**Nominal composition (at. %)**			**Phase**	**XRD analysis**				**Phase composition (at.%) measured by EDS**		
	**Co**	**Ni**	**In**		**space group**	**Lattice constant(nm)**					
						**a**	**b**	**c**	**Co**	**Ni**	**In**
26	28	24	48	$Co_{1-x}Ni_xIn_2$ (x = 0.612)	Fddd(70)	0.9416(3)	0.5279(2)	1.7736(3)	20.10(5)	16.82(5)	63.08(5)
				$Ni_{2-x}Co_xIn_3$ (x = 0.450)	$P\bar{3}m1(164)$	0.4402(3)		0.5316(3)	8.69(4)	32.99(4)	58.32(4)
				α-$Co_{1-x}Ni_x$ (x=0.200)	$P6_3/mm(194)$	0.2598(2)		0.4057(4)	82.21(5)	17.16(5)	0.63(5)
27	26	6	68	$Co_{1-x}Ni_xIn_2$ (x = 0.612)	Fddd(70)	0.9416(2)	0.5279(2)	1.7736(2)	13.11(3)	20.17(5)	66.72(3)
				In (Liquid)					0.93(5)	1.05(5)	98.02(5)
28	8	22	70	$Co_{1-x}Ni_xIn_2$ (x = 0.612)	Fddd(70)	0.9416(1)	0.5278(2)	1.7736(3)			
				$Ni_{2-x}Co_xIn_3$ (x = 0.450)	P-3m1(164)	0.4402(4)		0.5316(2)			
				In (Liquid)							
29	18	52	30	$Ni_{13-x}Co_xIn_9$ (x = 2.634)	C2/m(12)	1.4665(3)	0.8351(3)	0.8968(3)	10.91(5)	46.53(4)	42.56(4)
				ξ	$P6_3/mmc$ (191)	0.4171(3)		0.5112(2)	5.94(6)	55.83(5)	38.28(5)

				α-$Ni_{1-x}Co_x$ (x=0.500)	$Fm\bar{3}m$(225)	0.3518(5)			48.69(4)	49.12(4)	2.19(3)
30	20	45	35	$Ni_{13-x}Co_xIn_9$ (x = 2.634)	C2/m(12)	1.4663(2)	0.8352(3)	0.8968(4)	12.03(6)	47.28(7)	40.69(6)
				$Ni_{1-x}Co_xIn$ (x = 0.160)	P6/mmm (191)	0.5245(2)		0.4349(2)	7.43(5)	43.56(6)	49.01(6)
				α-$Ni_{1-x}Co_x$ (x = 0.600)	$Fm\bar{3}m$(225)	0.3519(5)			61.21(5)	37.51(5)	1.28(5)
31	14	66	20	Ni_3In	$P6_3$/mmc(194)	0.5543(3)		0.4227(5)			
				$Ni_{2-x}Co_xIn$ (x = 0.181)	$P6_3$/mmc (194)	0.4192(2)		0.5149(7)			
				α-$Ni_{1-x}Co_x$ (x = 0.400)	$Fm\bar{3}m$(225)	0.3516(4)					
32	12	58	30	$Ni_{2-x}Co_xIn$ (x = 0.181)	$P6_3$/mmc (194)	0.4192(2)		0.5149(7)			
				ξ	$P6_3$/mmc (191)	0.4171(3)		0.5112(2)			
				α-$Ni_{1-x}Co_x$ (x=0.500)	$Fm\bar{3}m$(225)	0.3518(5)					
33	20	34	46	$Ni_{1-x}Co_xIn$ (x = 0.160)	P6/mmm (191)	0.5246(3)		0.4348(2)	5.63(6)	43.68(5)	50.69(5)
				$Ni_{2-x}Co_xIn_3$ (x = 0.450)	$P\bar{3}m1$(164)	0.4402(3)		0.5316(3)	6.39(5)	34.92(4)	58.69(5)
				α-$Co_{1-x}Ni_x$ (x = 0.200)	$P6_3$/mm(194)	0.2598(5)		0.4057(4)	84.23(4)	13.88(4)	1.89(5)

© 2020 by the authors. Submitted for possible open access publication under the terms and conditions of the Creative Commons Attribution (CC BY) license (http://creativecommons.org/licenses/by/4.0/).
